# The Effect of Nano-Silica Surface Infiltration on Bond Strength of a Phosphate-Monomer–containing Composite Cement to Zirconia

**DOI:** 10.3290/j.jad.b3974633

**Published:** 2023-03-20

**Authors:** Chao Chen, Shuang Li, Meng En Ou, Yue Li, Qiang Sun

**Affiliations:** a Dentist, Center of Stomatology, China-Japan Friendship Hospital, Beijing, People’s Republic of China. Performed the experiments, wrote the manuscript, read and approved the final manuscript.; b Senior Engineer, Institute of Analysis and Testing, Beijing Academy of Science and Technology (Beijing Center for Physical and Chemical Analysis). Performed the experiments and data analysis, read and approved the final manuscript.; c Dentist, The 3rd Dental Clinic, Peking University School and Hospital of Stomatology, Beijing, People’s Republic of China. Data interpretation and analysis, read and approved the final manuscript.; d Dental Technician, Center of Stomatology, China-Japan Friendship Hospital, Beijing, People’s Republic of China. Technical assistance, read and approved the final manuscript.; e Associate Professor, Center of Stomatology, China-Japan Friendship Hospital, Beijing, People’s Republic of China. Study design, read and approved the final manuscript.

**Keywords:** zirconia, bond strength, nano-silica, adhesion.

## Abstract

**Purpose:**

To evaluate the bonding receptiveness of zirconia treated with nano-silica surface infiltration and the bond strength of composite cement after aging.

**Materials and Methods:**

Zirconia ceramic green bodies (Ceramill zolid, Amann Girbach) with dimensions of 10 x 10 x 4 mm were divided into three groups (n = 4): group C (control: no treatment after sintering), group S (sandblasted: 50-μm alumina airborne particle abrasion after sintering) and group N (nanosintered: infiltrated with nano-silica colloid, sintered, and then etched with hydrofluoric acid). Phase transformations were examined through X-ray diffraction (XRD). Composite resin (Filtek Z250, 3M Oral Care) was bonded to zirconia using the 10-MDP-containing composite cement Panavia F (Kuraray Noritake). The composite-cement/zirconia bond strength was immediately measured using the microtensile bond strength test (µTBS) as well as after three months of artificial aging in water (n = 20 microstick specimens/group). Failure mode patterns were examined using SEM.

**Results:**

The specimens of groups C and S, as tested by XRD, exhibited almost full tetragonal phases, while a small extent of tetragonal-monoclinic phase transformation (t→m) was observed for group N. Group N achieved the highest bond strengths (41.5 ± 8.6 MPa), which was significantly higher than that measured for groups C and S (p < 0.05). There was a significant drop in µTBS after 90 days of water storage for groups C and S. SEM revealed a decrease in the percentage of cohesive failure in groups N and S after water storage.

**Conclusions:**

Infiltrating zirconia with nano-silica is a reliable method to establish a strong and stable bond to zirconia. The combination of surface infiltration with nano-silica and application of a phosphate monomer-containing composite cement can significantly improve the composite-cement/zirconia bond strength.

Zirconia has gained popularity for a range of dental applications in recent years due to its attractive esthetics, chemical resistance, hardness, compression resistance, and biocompatibility.^8^ Zirconia is a multiphase crystal material, which can transform into different crystal phases under variable conditions of pressure and temperature.^8^ For example, pure zirconia exists in the stable monoclinic phase from room temperature to 1170°C and transforms into a tetragonal or cubic phase at higher temperatures. On the contrary, cooling zirconia to room temperature induces the opposite phase transition (t→m), resulting in the expansion of the crystal volume and crack propagation.^8,53^ This problem is overcome by yttrium-stabilized tetragonal zirconia polycrystals (Y-TZP), which contain yttrium trioxide (3 mol%) to prevent the crystal-phase transformation during the cooling process and forms a stable tetragonal phase zirconia at room temperature.^8,53^

In addition, stress-induced transformation further enhances the mechanical properties of Y-TZP. The change of external temperature and pressure causes transformation from the tetragonal to the monoclinic phase, which results in compressive stress at the surface and thereby increases the strength of zirconia.^10^ However, continuation of the t→m phase transformation of Y-TZP initiates surface flaws and the ejection of crystal grains, which leads to catastrophic effects and failure of the restoration.^17,53^

For silicon-based ceramics, hydrofluoric acid etching and the application of a silane coupling agent is a recommended method for adhesive luting using a composite cement.^12^ However, zirconia is highly inert and acid resistant due to its polycrystalline structure.^61^ Hence, the common clinical procedure of hydrofluoric acid etching does not produce the desired topographic features, unless high concentrations and temperatures are employed.^26^ Therefore, enhancing the bond strength of composite cement to the zirconia restoration may require additional mechanical conditioning, such as airborne particle abrasion.^49^ Airborne-particle abrasion is the most commonly used mechanical surface treatment method that can increase the mechanical retention of zirconia by cleaning and roughening the surface.^32^

The most commonly used material for airborne-particle abrasion is alumina (Al_2_O_3_) in the form of particles with a diameter ranging from 30 to 250 μm.^48^ The particle diameters and high air-abrasion pressure may affect the t→m crystal-phase transformation and long-term low-temperature degradation of Y-TZP.^32,56,64^ Airborne-particle abrasion may transform the surface grains from the tetragonal to the monoclinic phase, increasing the size of the grains, which subsequently produces surface compressive stress. This counteracts the flaw-induced reduction in strength.^10,23^ However, airborne-particle abrasion may cause microcracks on the surface, the propagation of which may reduce the strength and fracture toughness.^33^ To reduce damage to the surface, airborne-particle abrasion using 50-µm alumina particles with a pressure of 0.25 MPa or less is clinically recommended.^28^ A primer or an adhesive containing phosphate monomers – such as 10-methacryloyloxydecyl dihydrogen phosphate (10-MDP) – establishes chemical bonds with zirconia through ionic and hydrogen bonding.^22,31^ However, to obtain strong adhesion, the primer or adhesive containing 10-MDP should be supplemented with mechanical pretreatment of the zirconia surface.^16,52,65^

Although using resin-bonded fixed dental prostheses (RBFPDs) with zirconia-ceramic single retainers to replace an anterior tooth has yielded high survival rates,^30,40^ there is disagreement in the literature regarding the survival rate of zirconia RBFPDs in the posterior region. Rathmann et al^42^ reported a high incidence of chipping and debonding using this technique, and other authors found a lower 10-year probability of survival (12%) when using zirconia RBFPDs in the posterior region.^55^ Currently, published work on clinical long-term resin bonding data using partial-coverage high-strength ceramic or monolithic zirconia restorations is scarce.^11^ The most common complication remains the debonding issue, which can be improved either by the addition of oral and buccal wings^1,63^ or by a more effective bonding protocol.^28^ In contrast, monolithic IPS e.max lithium-disilicate glass-ceramic (Ivoclar Vivadent; Schaan, Liechtenstein) partial coverage restorations have exhibited a higher survival rate (from 95.6% to 100%). The most common complication of this approach is bulk fracture or large chips rather than debonding.^24,36^ Therefore, the development of zirconia bonding is likely to further improve the prognosis of these restorations. In addition, this approach may also reduce the degree of complexity of preparation by omitting some delicate preparation geometries, such as wings, proximal boxes, and pinholes.

To improve the composite cement bond to zirconia, a range of mechanical and chemical surface treatment techniques have been studied, including tribochemical silica coating,^45^ experimental hot etching solution,^13^ selective infiltration etching,^37^ laser irradiation,^7^ and chemical vapor deposition.^51^ Various methods have been investigated to integrate silica into the surface of zirconia, which can then be chemically bonded by resin via a silane-based coupling agent. Examples of such agents include silica-based nanocoating by magnetron sputtering, in which thin SiO_2_ films are deposited on the surface of Y-TZP blocks using a magnetron-sputtering method of physical vapor deposition.^54^ Another silica nanoparticle deposition method utilizes two alkoxide organic precursors to enhance the deposition of a SiOx reactive layer onto Y-TZP.^44^ The adhesion achieved between composite cement and zirconia by these two methods was similar to that of air abrasion followed by primer application.^44,54^ Silica coating can also be performed by a sol-gel process,^35^ but this is impractical in clinical practice due to long deposition times and weakening of resin bonding compared to sandblasting.^35^ The aim of the present study was to evaluate the bond strength of composite cement to zirconia pre-treated with different conditioning methods, and the effect of water-storage aging on bond strength. The present research tests the hypothesis that phosphate monomer-containing composite cement bond strengths to Y-TZP can be improved using the infiltrating nano-silica technique, compared to other surface treatments such as airborne-particle abrasion or 10-MDP monomer alone.

## Materials and Methods

### Preparation of the Zirconia Specimens

**Fig 1:**
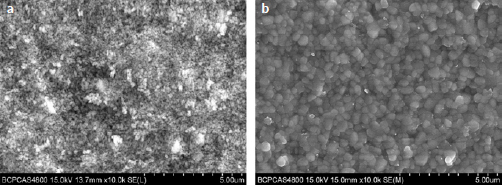
a) Image of pores between grains of the zirconia green body. b) The grains of sintered zirconia coalesce tightly and the pores disappear.

The present study used a zirconia green body (Ceramill zolid, Amann Girbach; Pforzheim, Germany) with small pores with a grain diameter of ~1 μm. During the sintering process, the growth of crystal grains leads to gradual shrinkage of the pores and tightening of the grain boundaries, as is shown in Fig 1. The present study investigated zirconia surface treatment by infiltrating nano-silica into the zirconia green body. Colloidal silica containing silicon dioxide with a diameter of 12 nm was applied to the surface of the zirconia green body. Under negative pressure, the nano-silica infiltrates the pores, and is then sintered to leave the nano-silicon on the surface. Finally, the silicon dioxide on the surface can be etched by hydrofluoric acid, forming inter-grain nanopores and facilitating the infiltration and interlocking of a phosphate monomer-containing composite cement.

A total of 12 pre-sintered yttria tetragonal zirconia polycrystal (3Y-TZP 3 mol% yttria content) bar-shaped specimens (10 x 10 x 4 mm) were fabricated by cutting zirconia milling blocks (Ceramill zolid, Amann Girbach) using a cutting machine (Ceramill Motion 2, Amann Girbach) under water cooling. All the fabricated specimens were measured using an electronic vernier caliper (Deli, China), and randomly divided into three experimental groups (n = 4 each). In the control group (group C), specimens were sintered without any further treatment. For sintering, the furnace was heated to 1450°C at a rate of 8°C/min, and the temperature was held at 1450°C for 2 h (the moment of sintering) before cooling at a rate of 20°C/min. In the experimental group S, the specimens were sintered and then airborne-particle abraded using 50-μm aluminum oxide particles (Renfert; Hilzingen, Germany) at 0.2 MPa pressure at a distance of 10 mm for 10 s. The experimental (group N) specimens were infiltrated with colloidal silica (LUDOX HS-40; St Louis, MO, USA). The colloidal solution was applied on the surface of zirconia disks using a small brush, dried under vacuum conditions (0.1 MPa; 5 min) (Vacuum Pump 2C, Vacuubrand; Wertheim, Germany), and sintered. The infiltration agent was dissolved in a 5% hydrofluoric acid liquid (Xilong, China) bath for 10 min. All the specimens were steam cleaned and air dried.

### X-ray Diffraction (XRD)

The phase ingredients of specimens were identified using a monochromatic Cu-Kα radiation X-ray diffractometer (Bruker D8 Advance, Bruker AXS; Karlsruhe, Germany). The surface of the specimens was scanned at 40 mA and 40 kV between 3 and 90 2Ɵ (degrees), with a step size of 0.02. The XRD spectra of specimens were analyzed using the corresponding computer software (Eva, Bruker AXS). The relative amount of transformed monoclinic phase (X_m_) on the Y-TZP surfaces was calculated using the following equation:


$$X_{m}=\left[I_{m}(-111)+I_{m}(111)\right]/\left[I_{m}(-111)+I_{m}(111)+I_{t}(101)\right]$$

as described by Garvie and Nicholson,^22^ where I_m_ (−111), It (101) and _m_ (111) are the intensities of the peaks around 28, 30, and 31 degrees, respectively.

### Adhesive Luting Technique

Composite resin (Filtek Z250, shade A1, 3M Oral Care; St Paul, MN, USA) specimens (10 x 10 x 4 mm) were prepared in a transparent plastic mold and light polymerized from four different sides for 15 s per side using an LED curing light (Satelec Mini LED, KaVo Dental; Biberach, Germany). The light intensity was 800 mW/cm^2^ and the distance from the light source was 5 mm. The resin disks were polished using silicon carbide papers (grit # 120, 240, 360, 480, 600, and 900) (Panda; Beijing, China) in ascending order for 30 s without water. The composite resin specimens were then sonicated in deionized water for 10 min and stored in distilled water (at 37°C) for three months prior to being bonded to the zirconia surface. A phosphate-monomer–containing composite cement (Panavia F, Kuraray Noritake; Tokyo, Japan) was used to bond the composite disks to the zirconia substrate. For all three groups, equal amounts of ED Primer II A&B (Kurarary Noritake) were mixed and applied to the composite disks. After waiting for 30 s, the disks were gently air dried. Further, pastes A&B were mixed for 20 s, and the mixture was applied to the zirconia disks. This was followed by seating each disk on top of the resin substrate with 50 N pressure for 60 s using a special loading device (force gauge; Handpi, China). Any excess cement was wiped off. Finally, the specimens were light polymerized at four different locations for 60 s each (Satelec Mini LED, KaVo).

### Microtensile Bond Strength (µTBS) Test

Each composite-zirconia block was vertically sectioned into sticks (1 mm^2^ cross section, 6 mm long) using a diamond-coated disk of a precision cutting device and copious amount of water (Isomet 1000, Buehler; Lake Bluff, IL, USA). The microsticks were carefully examined using a stereomicroscope (Olympus; Tokyo, Japan) and only structurally intact, crack-free sticks were selected (20 microsticks for each group). The length of the bonding cross-section of each stick was measured using an electronic vernier caliper (Deli). Each microstick was bonded to a stainless-steel attachment unit using instant glue (Loctite 495, Henkel; Düsseldorf, Germany), positioning the composite-cement/zirconia interface in the free space between two parts of the attachment unit. To calculate the composite cement-zirconia µTBS (MPa), an axial load was applied to the bonded interface (1 mm^2^) using a universal testing machine (Zwick/Roell Z020; Ulm, Germany) at a crosshead speed of 1 mm/min until failure of the composite-zirconia interface. The load cell (200 N) was calibrated using standardized loads. Failure load was calculated using the supplementary computer software. Using the microtensile bond strength (µTBS) test, the composite cement-zirconia bond strength was evaluated immediately) and after three months of water storage (n = 20 microsticks/group).^47^

### Analysis of Failure Mode

The fractured microsticks were ultrasonically cleaned, dried, mounted on metallic stubs, gold sputter-coated and examined under a SEM (S-4800, Hitachi; Tokyo, Japan) at a magnification of 100X. The mode of failure was classified as either cohesive failure in composite cement if the crack originated outside the bonded interface, or interfacial failure if the crack travelled along the zirconia-composite cement interface. Mixed failure was defined as a combination of the above two modes.^3^

### Statistical Analysis

All data were evaluated for normal and equal distributions (Kolmogorov-Smirnov and Levene’s tests, respectively). Two-way ANOVA, with a main effect of group variable (zirconia surface treatment, three levels) and between factors (aging or not, two levels), was used to analyze the data (α = 0.05). A Bonferroni post-hoc test was utilized for pairwise comparisons (α = 0.05).

## Results

### XRD Analysis

**Fig 2:**
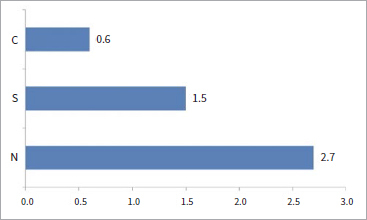
Relative amount of transformed monoclinic phase (X_m_) (%).

**Fig 3:**
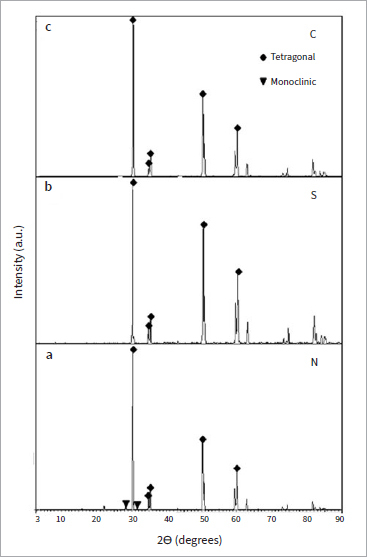
Comparison of the XRD spectra of various study groups. XRD revealed a small monoclinic peak at 28 degrees in group N (a). The monoclinic peaks were reduced to nearly zero in groups S (b) and C (c).

When comparing the X_m_ (%) values, groups C and S exhibited almost full tetragonal phases with a relative amount of transformed monoclinic phase (X_m_) values of 0.6% and 1.5%, respectively (Fig 2). XRD revealed that the monoclinic peaks decreased to nearly zero in groups C and S (Fig 3). In contratst, after infiltration with nano-silica, sintering, and etching, a small monoclinic peak appeared at 28 degrees. The relative amount of transformed monoclinic phase (X_m_) on the Y-TZP surfaces of group N was 2.7%, suggesting that the infiltration of silica had little effect on the X_m_ values.

### µTBS Test

Data analysis revealed a significant difference in mean µTBS between the three tested groups (p < 0.001). The mean µTBS of the as-sintered group (C) was 22.7 ± 4.9 MPa, and was significantly higher (S: 31.3 ± 10.3 MPa) in the airborne-particle abrasion group and the infiltration group (N: 41.5 ± 8.6 MPa) (Table 1). Overall, three months of water storage significantly affected the bond strength to zirconia (p < 0.05). After three months of water storage, the mean µTBS of groups C, S, and N decreased to 13.6 ± 4.7 MPa, 26.2 ± 6.2 MPa, and 37.6 ± 7.0 MPa, respectively (Table 1). For group N, the initial µTBS decreased but not significantly, unlike the as-sintered and airborne-particle abraded specimens, which demonstrated a significant reduction in µTBS after water storage.

**Table 1:** Mean microtensile bond strength to zirconia (MPa ± SD) and mode of failure of test groups before and after three months of water storage

Test group	Immediately tested		3-month water storage		µTBS decrease
	µTBS	Failure type	µTBS	Failure type	
Group C	22.7 ± 4.9^A^	90% interfacial	13.6 ± 4.7^a^	90% interfacial	40%
Group S	31.3 ± 10.3^B^	80% cohesive	26.2 ± 6.2^b^	60% cohesive	16%
Group N	41.5 ± 8.6^C^	90% cohesive	37.6 ± 7.0^c^	80% cohesive	9%

Different superscript uppercase or lowercase letters indicate a statistically significant difference (p < 0.05) in µTBS between groups.

### SEM Analysis

**Fig 4:**
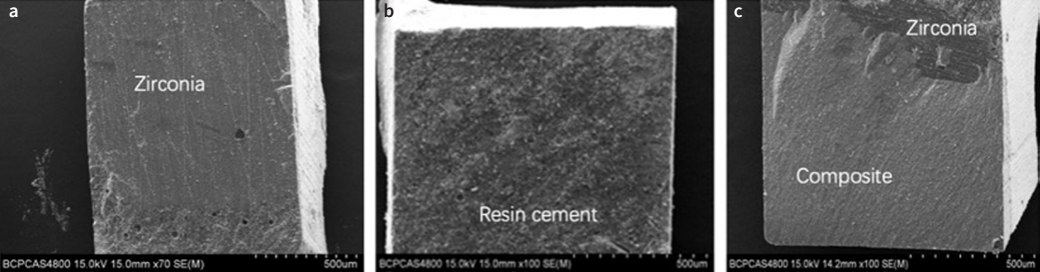
SEM images of the fractured microsticks. (A) Interfacial failure: the surface of zirconia was almost completely exposed. (B) Cohesive failure in composite cement: the surface of zirconia is covered by a layer of composite cement. (C) Mixed interfacial and cohesive failures where a part of the surface of zirconia was exposed and a part of the surface remained covered by composite resin.

The SEM analysis of the fractured microsticks revealed predominantly interfacial failure for the as-sintered specimens (group C), as most of the surface area of zirconia was exposed after fracture, indicating a weak area along the bonding interface (Fig 4a). In contrast, specimens of group N predominantly demonstrated cohesive failure in the composite cement, where the crack originated outside the composite cement/zirconia interface (Fig 4b).^3,5,38^ The group S specimens exhibited predominantly mixed interfacial and cohesive failures at the resin-zirconia interface (Fig 4c). After three months of water storage, the occurrence of mixed failures had increased in groups N and S; however, the failure pattern percentages of group C specimens did not change (Table 1).

## Discussion

Selective infiltration etching (SIE) is a relatively new technique for zirconia surface treatment that establishes strong, stable bonding.^4,5^ During the sintering process, zirconia undergoes heat-induced maturity^5^ and generates stresses between the grain boundary regions and diffusion of small dopants, such as Si or Ti, through the grain boundaries.^15,41,58^ Although HF etching of the silicon on the surface of zirconia establishes micropores, this approach is complex, sensitive,^2^ and requires further investigations before it can be applied clinically.^37^This study used the infiltration of nano-silica into the zirconia green-body surface, which is different from SIE technology. Due to the high porosity of green-body zirconia, nano-silica infiltrated into the pores before sintering and surface etching.

In the present study, the X values in groups C and S ranged from 0.6–1.5%, and XRD patterns revealed that monoclinic peaks decreased to nearly zero. The monoclinic phase of group N was 2.7%, and a small monoclinic peak appeared at 28 degrees in the XRD pattern. These findings suggested that the airborne-particle abrasion conducted in our study had little effect on the t→m phase transformation, which disagrees with previous studies.^39^ The conflicting results may be attributed to the different methodologies used by other groups, such as using a different diameter of Al_2_O_3_ particles and different pressures during airborne-particle abrasion. Kosmac et al^31^ abraded specimens with 110-μm Al_2_O_3_ particles at 4 bar pressure, resulting in the highest monoclinic phase. In contrast, the present study used 50-μm Al_2_O_3_ particles at a lower pressure (0.2 MPa). In addition, we observed only a small peak that is characteristic of the monoclinic phase in the XRD patterns of group N, suggesting that the infiltration method affected the t→m transformation. Therefore, flexural strength testing of the infiltrated, etched specimens is needed to evaluate the effects of this method on the structural integrity of the Y-TZP specimens.

Commonly used methods to measure bond strength include tensile and shear bond strength tests.^25^ Considering that shear stress does not localize at the interface of the bond, uneven distribution of de-bonding stress at the interface may lead to cohesive failure^20^ and erroneous interpretation of the actual bond strength.^14^ Microtensile bond-strength testing was first introduced in 1994 by Sano et al,^57^ who demonstrated that microtensile failure occurs at the bonded interface. Microtensile bond strength testing can more accurately evaluate the actual bonding and failure effects.^50,57,59^ Meanwhile, the bonding area of the microstick specimen is small, which ensures fewer structural defects and results in a lower scattering of the data.^2^ In addition, the microtensile bond strength test can better reflect the effects of aging.^47^ Due to the small bonding area being stored in water, the bonded surface can be more fully hydrolyzed and aged, and it is more sensitive to water storage aging.^6,47^ However, creating and processing the small microtensile bond strength test specimens is technique sensitive and time consuming.^2^ In the present study, the treatment of the group N specimens created three-dimensional inter-grain nanopore structures that facilitated the penetration of composite cement and adhesion with zirconia. The retention effects of infiltration surface treatment were better than that of airborne-particle abrasion, resulting in higher µTBS for group N.

In the complex oral environment, dynamic changes in salivary composition, temperature, and masticatory stresses on restorations may affect bond strength. Therefore, to evaluate the long-term clinical behavior, it is important to assess the long-term bond strength, where the bonded specimens are subjected to different aging conditions that simulate clinical situations.^18^ Some commonly used aging methods include water storage and thermocycling.^47^ The principle of water storage aging is water uptake and hydrolytic degradation. Similarly, thermocycling in-vitro simulates in-vivo hydrothermal aging. Temperature changes induce repetitive contraction-expansion stresses that occur at the bonded interface or inside the materials, which may exert a significant influence on bond strength.^47^ A meta-analysis of microtensile bond strength testing of stick-shaped specimens (~ 1mm^2^) following aging via water storage (90 days or more) showed a significant reduction in bonding strength.^47^ Another meta-analysis showed that simple water-storage aging is better than thermocycling, since it can more accurately evaluate the durability of the bond between resin and zirconia.^27^ The present study conducted three months of water-storage aging, which diminished the bond strength of all the three groups. The µTBS of groups C and S decreased significantly, while the percentage of µTBS decrease in group S was lower than that of group C, suggesting that adhesion to zirconia depends on both chemical and micromechanical bonding. These findings are consistent with previous studies.^16^ Group N had better anti-aging effects than groups C and S, as the µTBS of group N demonstrated a non-significant decrease. In addition, the group N specimens displayed three-dimensional inter-grain nanopore structures that facilitated the penetration and retention of composite cement. The infiltration surface treatment produced better retention effects than did airborne-particle abrasion, resulting in the enhancement of anti-aging effects for group N.

**Fig 5:**
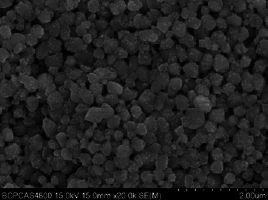
Inter-grain nanopores formed after the infiltration of nano-silica, which was etched by hydrofluoric acid.

Group C mainly exhibited interfacial failure patterns, whereas group N demonstrated mostly cohesive failure patterns. Predominantly mixed interfacial and cohesive failure patterns were found in group S. These findings were related to the retentive surface (Fig 5) created by nano-silica infiltration, which resulted in the creation of nano-mechanical retention with the phosphate monomer-containing composite cement used. After three months of water storage, the proportion of mixed failure pattern of groups S and N increased, suggesting hydrolytic degradation of the bonding interface.^9^ However, group N still predominantly exhibited cohesive failure after water storage, indicating that the interlocking effect between the three-dimensional inter-grain nanopores and resin resisted hydrolysis.

Kern et al^32^ reported that a composite cement containing phosphate monomer can establish a durable bond with zirconia. In that study, two chemically cured phosphate monomer-containing composite cements – Panavia Ex with MDP (Kuraray Noritake) and Panavia 21 Ex (Kuraray Noritake) – and one composite cement without phosphate monomer (bis-GMA) were compared. The results showed that Panavia Ex and Panavia 21 Ex can resist artificial aging and maintain high bond strength.^30,63^ However, as previously mentioned, the composite cement containing phosphate monomer should be combined with mechanical surface pretreatment to establish stable bonding.

It is well known that hydrofluoric acid (HF) can dissolve the glass-matrix phase by reacting with silicon dioxide.^60^ The creation of microporosities on glass-matrix ceramics using HF has been the standard procedure for adhesive cementation of porcelain restorations.^19^ There are many brands of ceramic etchants in dentistry.^34^ The concentration of HF ranges from ~5% to ~10%; most HF etchants are in a gel base, which facilitates manipulation in the clinical setting.^34^ The HF etchant used in our study contained 5% HF in solution. In-vitro, infiltrating nano-silica can be dissolved by immersing ceramic disks in an ultrasonic bath with 5% HF solution for 10 min.^4^ The HF recommendations for use in restorative dentistry have been reviewed elsewhere.^46^

The present findings may guide clinicians to consider the application of an infiltrating nano-silica solution to enhance bond strength and reliability, particularly in cases of restorations with poor retention. Although this study showed that infiltrating nano-silica surface treatment can improve the bond strength between Panavia F and zirconia, further clinical trials are needed to validate our findings. At the same time, the impact of infiltration treatment on the strength of zirconia also requires further investigation.

## Conclusion

Infiltrating nano-silica is a reliable method that can establish a strong, stable bond to zirconia substrates when combined with Panavia F (Kuraray Noritake). Airborne-particle abrasion combined with Panavia F can also improve the resin-zirconia bond strength, but the mean bond strength may decrease after aging. Therefore, using Panavia F alone may not be a reliable bonding method.

## Clinical relevance

Nano-silica surface infiltration treatment is a practical and reliable method that can establish a strong, stable bond to zirconia when combined with composite cement.
